# An analysis of the factors influencing engagement metrics within the dissemination of health science misinformation

**DOI:** 10.3389/fpubh.2025.1571210

**Published:** 2025-06-06

**Authors:** Ruofei Chen, Guoyong Chen, Li Zhang, Ruiqian Xie, Ruopeng Chen

**Affiliations:** ^1^China Health Education Center, Beijing, China; ^2^Henan Provincial Veterans Hospital, Xinxiang, Henan, China

**Keywords:** health misinformation, regression analysis, engagement metrics, dissemination, non-parametric testing

## Abstract

**Objective:**

The proliferation of health misinformation on social media platforms presents a significant challenge.

**Methods:**

Data were collected from WeChat, video websites, and Weibo in November 2024. A total of 109 health misinformation samples were selected using our team’s “Health Misinformation Screening Criteria.” This study analyzes the activity and influencing factors of this misinformation. Activity indicators, including reads (views), comments, reposts, and likes, were weighted based on communication theory principles. A combined weighting method, using the entropy weight method and the analytic hierarchy process (AHP), was employed, followed by sensitivity analysis to determine activity levels. Non-parametric tests and negative binomial regression models were used to analyze the key influencing factors of misinformation activity.

**Results:**

WeChat exhibited the highest proportion of health misinformation (44.95%), with the majority originating from individual authors (55.96%), and primarily positive sentiment (37.61%). Misinformation related to disease prevention and control was most prevalent (54.13%), with declarative sentences being the most common tone (55.04%). Significant differences in propagation activity were observed, with WeChat exhibiting the highest activity, followed by video websites, and then Weibo. Misinformation with negative sentiment had significantly higher interactivity than neutral and positive content. Misinformation published by institutional authors was more likely to spread due to their authoritative advantage. Negative binomial regression analysis indicated that the disease prevention and control theme, three types of tone (interrogative, declarative, and exclamatory sentences), positive sentiment, and institutional authors significantly influenced misinformation activity (*p* < 0.05).

**Conclusion:**

By illustrating misinformation cases and stratified prevention strategies, this study reveals the key roles of themes, expression forms, sentiment, and publishing entities in the spread of health misinformation. It provides foundational data and theoretical support for targeted prevention, follow-up research, and the formulation of relevant management strategies, promoting a comprehensive governance model of “platform technology interception - science education prevention - misinformation source management.”

## Highlights

- In light of the burgeoning evolution of social media, this study meticulously examines the dynamics of health-related misinformation, a subject that has been infrequently explored in previous research within this specific field.
- Amidst significant shifts in information dissemination patterns driven by social media, the spread of health-related misinformation reveals novel characteristics and trends. This research adeptly identifies this emerging focus, setting the stage for further comprehensive investigation into its dissemination mechanisms and influencing factors, thereby addressing a notable gap in the academic literature from this vantage point.

## 1 Introduction

The advent of the internet and the proliferation of social media have fundamentally altered information-seeking behaviors. A growing segment of the population now utilizes the internet to access health information, with social media platforms emerging as significant channels for the dissemination of health-related content. However, this evolution has also facilitated the propagation of health misinformation. The diminished influence of expert voices on social media, compounded by audience-specific psychological factors and varying levels of health literacy, contributes to the widespread dissemination of unsubstantiated health claims. Furthermore, a lack of specialized knowledge hinders the public’s ability to critically evaluate information, thereby exacerbating the spread of health misinformation. The circulation of health misinformation on social media not only misleads the public but also potentially undermines social order. Historically, the term “rumor” first appeared in the Han Dynasty, referring to songs and praises. Ancient interpretations of rumors encompassed both unfounded hearsay and folk songs or proverbs critiquing political affairs. Allport (1) provided an early definition of rumor, characterizing it as “a specific proposition or statement for belief, transmitted from person to person, usually by word of mouth, without secure standards of evidence being present.” Kapferer (2) expanded the definition, defining rumors as “information that appears and circulates in society, which has not been officially confirmed or has been refuted by official sources.” Generally, health misinformation is defined as unverified or erroneous health information disseminated through social media, online forums, and other channels, encompassing areas such as food safety, nutritional supplements, and disease prevention.

## 2 Methods

### 2.1 Health misinformation screening criteria

This study utilized the “Wenhai Big Data Platform,” a technology transfer platform from the Chinese Academy of Sciences (Zhongke Wenge), to conduct screening based on our research team’s “Health Science Communication Keyword Table.” Information was classified as “typical health science communication misinformation” if it met three primary criteria (3): first, the information demonstrated scientific inaccuracy, encompassing factual errors, contradictions of scientific consensus, and a lack of substantiation from official sources and scientific literature. Second, the information exhibited characteristics of a propagation hotspot, as indicated by metrics such as the number of websites/media disseminating the information, view counts, reposts (or shares), comments, and likes, which are critical for evaluating the dissemination rate and extent of health science communication misinformation. Third, the information presented significant harm risks, including potential threats to public health and safety, adverse impacts on economic and social development, and risks to social harmony and stability. It is important to note that scientifically accurate debunking information was excluded from the classification of health science communication misinformation.

### 2.2 Data sources

The data samples for this study were sourced from WeChat, video websites, and Weibo. A non-probability sampling method was employed, utilizing web crawler tools to collect all data from November 1, 2024, to November 30, 2024, using “health science communication keywords.” Initial screening of over 2,000 suspicious samples was conducted, followed by the application of computer logic to eliminate duplicates and irrelevant content, such as fiction, resulting in a selection of over 391 suspicious samples. These samples were then manually assessed by professionals, based on the health science communication misinformation screening criteria, to identify 266 suspicious samples. Finally, these samples underwent categorization, review, and validation by academic committee experts or experts in relevant fields, and through literature verification, 109 samples were ultimately confirmed.

### 2.3 Inclusion and exclusion criteria for materials

Inclusion criteria: ① information verified as misinformation; ② information pertaining to health science popularization. Exclusion criteria: ① duplicative materials; ② information irrelevant to health science popularization.

### 2.4 Theoretical framework

This study operationalizes the activity levels of the sample. The activity level of health science popularization misinformation serves as the primary metric for assessing their dissemination extent and rate. This study is grounded in four communication theories: uses and Gratifications Theory, Social Presence Theory, Six Degrees of Separation Theory, and Communication Effects Theory. It posits that the indicators influencing the activity level of health science popularization misinformation include the number of comments, reposts, views, and likes.

#### 2.4.1 Uses and gratifications theory

Audiences fulfill their needs through the active selection of media content. Interactive behaviors (e.g., reposts and comments) reflect higher-order need satisfaction. McQuail (4) further posits that interactivity indicators (e.g., comments and reposts) are the core dimensions for measuring audience “participatory communication,” directly impacting the secondary dissemination and social diffusion of information.

#### 2.4.2 Social presence theory

Interactive behaviors on social media (e.g., comments and likes) can enhance users’ emotional connection with information, thereby forming a “virtual presence,” and consequently improving communication effects. Short et al. (5) emphasize that the number of comments and reposts are key indicators for measuring the “social stickiness” of information, directly reflecting users’ recognition and willingness to disseminate the information.

#### 2.4.3 Six degrees of separation theory

The dissemination of content is critical for information to traverse social networks, consistent with the “weak ties theory.” Conversely, comments signify users’ in-depth cognitive processing of information. These two elements constitute the core activity factors. Watts (6) empirically demonstrated that information accompanied by user comments exhibits a higher probability of re-dissemination, thereby generating a “ripple effect of propagation.”

#### 2.4.4 Propagation effect theory

Within the domain of propagation effect research, Lazarsfeld et al. (7) two-step flow theory posits that information propagates from mass media to opinion leaders, and subsequently from opinion leaders to the general audience. The number of views serves as a proxy for the reach of health science misinformation during its initial dissemination phase. A substantial audience exposure to health science misinformation suggests the potential for its entry into the propagation chain.

### 2.5 Weighted analysis

Building upon the aforementioned theoretical frameworks, this study exclusively considers activity, excluding other variables. Employing the entropy weight method and the Analytic Hierarchy Process (AHP), a combined weighting approach is applied to the four indicators: views, comments, shares, and likes. The AHP process involves a panel of five health communication experts constructing a judgment matrix for the four indicators, grounded in industry consensus. Subsequently, the weights derived from the entropy weight method and AHP are normalized to compute a comprehensive weight (each contributing 50%). Sensitivity analysis is then performed to ascertain the specific impact of these weights on the activity assessment outcomes.

### 2.6 Data categorization and organization

#### 2.6.1 Thematic analysis of health science rumors

The sample data, derived from thematic keywords and content analysis, were stratified into three primary categories: healthy lifestyle, disease prevention, and traditional Chinese medicine (8).

#### 2.6.2 Emotional valence of health science rumors

The emotional valence of health-related rumors was classified into three distinct categories (9): neutral, positive, and negative.

#### 2.6.3 Linguistic tone of health science rumors

To evaluate the influence of linguistic tone on the perceived credibility of health-related rumors, the sample data were analyzed and categorized into four types based on their linguistic characteristics: declarative sentences, imperative sentences, exclamatory sentences, and interrogative sentences (10).

#### 2.6.4 Rumors classification of health misinformation authors

Authors were categorized into three groups: ① institutions; ② private individuals; and ③ corporations.

### 2.7 Statistical analysis

Data entry was performed using Epi Data 3.1, followed by statistical analysis using SPSS 27.0. A composite weighting methodology was employed to determine the comprehensive weights (each contributing 50%) of the four indicators influencing the activity of health science popularization rumors. Sensitivity analyses were subsequently conducted to validate the explanatory power of these weights on activity. Non-normally distributed measurement data are presented as median (interquartile range) [*M* (Q1, Q3)], and the Kruskal-Wallis H test was employed for intergroup comparisons. Given the data’s non-normal distribution and the nature of count data, a negative binomial regression analysis model was utilized. Statistical significance was established at *p* < 0.05.

## 3 Results

For this investigation, a four-indicator matrix was formulated, grounded in industry consensus and validated by five health communication experts. Utilizing a hybrid approach, the entropy weight method and analytic hierarchy process (AHP) were employed to ascertain the weights of reposts, reads, likes, and comments (Table 1). Following this, the weights derived from both the entropy weight method and AHP were normalized, and comprehensive weights were computed, with each method contributing 50%. The resulting weights for reposts, reads, likes, and comments were 0.4115, 0.2365, 0.174, and 0.178, respectively. A sensitivity analysis was then performed to assess the influence of these weights on the evaluation outcomes.

**Table 1:** Comparative analysis of results utilizing the analytic hierarchy process (AHP) and entropy weighting method.

AHP hierarchical analysis					Entropy weight method
Variable	Eigenvectors	Percentage weighting (%)	The dominant eigenvalue	Confidence Interval (CI)	Percentage weighting (%)
Weights for comments	0.387	9.67	4.043	0.014	26.0
Weights for likes	0.387	9.67			25.1
Weights for reads	1.007	25.165			22.1
Weights for reposts	2.22	55.495			26.8

### 3.1 Univariate sensitivity analysis

Univariate sensitivity analyses were performed to assess the impact of individual indicators on the activity score of health-related science communication rumors. This involved independently varying the weights of each indicator by ±10 and ±20%. The findings indicated that the weight assigned to reposts exerted the most significant influence on the activity score. Specifically, a 20% increase in the weight of reposts to 0.4938 resulted in an average 15% increase in the activity score. Conversely, a 20% reduction in the weight of reposts to 0.32109 led to an average 12% decrease in the activity score. These results underscore the critical role of reposts in evaluating the activity of health-related science communication rumors, with minor weight adjustments yielding substantial variations in the activity score. The activity score exhibited relatively less sensitivity to alterations in the weight of views. A 20% increase in the weight of views to 0.2838 was associated with an average 8% increase in the activity score, while a 20% decrease to 0.18109 resulted in an average 7% decrease. The effects of weight changes for likes and comments on the activity score were comparable. A 20% increase in the weight of likes to 0.2088 led to an average 6% increase in the activity score, and a 20% decrease to 0.13109 resulted in an average 5% decrease. Similarly, a 20% increase in the weight of comments to 0.2136 was associated with an average 7% increase in the activity score, and a 20% decrease to 0.1424 resulted in an average 6% decrease.

### 3.2 Multivariate sensitivity analysis

Multivariate analyses were performed to assess the impact of manipulating the weights of multiple factors. In the first scenario, the weights of both shares and views were increased by 10%, while the weights of likes and comments were decreased by 10%, which resulted in an average increase of 8% in the activity score. In the second scenario, the weight of shares was increased by 20%, the weight of views was decreased by 10%, the weight of likes was increased by 10%, and the weight of comments was decreased by 20%, leading to an average increase of 10% in the activity score. The results of the multivariate sensitivity analysis suggest that the impact of the synergistic changes in the weights of different indicators on the activity score is not a simple superposition of the effects of each single factor, but rather involves complex interactions. Overall, the weight of shares maintains a dominant influence on the activity score in the multivariate scenario.

### 3.3 Comprehensive sensitivity assessment

Through both univariate and multivariate sensitivity analyses, it was determined that the evaluation results of health science popularization rumor activity are most sensitive to the weight of shares. The weights of views, likes, and comments showed relatively lower sensitivity; however, their importance in the evaluation system is supported by the data.

### 3.4 Key findings

A total of 109 health-related science communication rumors were identified, primarily originating from WeChat, video websites, and Weibo (Table 2). Due to data attrition during statistical analysis, Weibo exhibited a reduced sample size relative to the other two platforms. WeChat comprised the largest proportion of the sample (44.95%). Regarding activity levels, WeChat demonstrated the highest engagement with health-related science communication rumors, followed by video websites, with Weibo showing the lowest activity. Statistically significant differences (*p* < 0.05) were observed in the activity levels of health-related science communication rumors across different platform sources (11). Among the thematic categories, disease prevention and control constituted the largest sample (54.13%), indicating a significant focus on this topic within the study. However, no statistically significant differences were found in the activity levels across different themes (*p* > 0.05) (12). Concerning the tones employed in health-related science communication rumors, declarative sentences accounted for the largest proportion, with 60 samples (55.04%), suggesting their frequent utilization in the study. Although interrogative sentences exhibited a higher median, the activity level differences among different tones were not statistically significant (*p* > 0.05). Positive sentiment was the most prevalent emotional form, with 37.61% of the samples, suggesting its frequent occurrence in the study. Negative sentiment forms demonstrated significantly higher activity levels compared to neutral and positive sentiment forms, as evidenced by both the median and rank mean, which may suggest that negative sentiment content is more likely to attract audience attention or generate higher interaction. Conversely, positive sentiment forms exhibited the lowest activity levels, possibly because individuals may not actively share and disseminate positive sentiment content as readily as they would with more stimulating or concerning information, leading to lower activity. Statistically significant differences were observed in the activity levels of health-related science communication rumors across different emotional forms (*p* < 0.05). Regarding the sources of health-related science communication rumors, private authors accounted for the largest sample size, reaching 55.96%, indicating their significant share in the statistics. In terms of activity levels, institutional sources exhibited the highest activity, while private sources showed the lowest, with corporate sources falling in between. This may be related to factors such as resources, influence, and participation methods of different author types. Institutions may possess more resources and channels to promote content, thereby achieving higher activity; while private authors may have relatively lower and more dispersed activity due to individual differences. Statistically significant differences were observed in the activity levels of different authors (*p* < 0.05).

**Table 2:** The daily engagement metrics of different types of variables.

	Type	1-day activity [*M*(25%, 75%)]	Rank mean	*H*	*P*
Different platforms	WeChat	7.00(1.00, 46.00)	43.71	24.540	0.000
	Video platforms	58.00(23.00, 119.00)	71.33		
	Weibo	3.00(1.00, 16.00)	36.50		
Different themes	Healthy lifestyle	10.00(1.00, 41.00)	42.90	2.836	0.242
	Disease prevention	34.00(10.50, 127.00)	59.09		
	Traditional Chinese medicine	29.00(1.00, 110.50)	51.99		
Different mood types	Imperative	42.00(20.00, 433.00)	68.44	6.780	0.079
	Declarative	14.00(1.00, 67.00)	48.08		
	Interrogative	51.00(8.00, 88.00)	62.87		
	Exclamatory	44.50(21.00, 76.00)	59.50		
Different emotional forms	Neutral	8.00(2.00, 68.00)	48.11	10.488	0.005
	Positive	22.00(1.00, 56.00)	49.68		
	Negative	58.00(21.00, 152.50)	70.47		
Different author	Institution	41.00(21.50, 430.50)	70.25	7.641	0.022
	Private	14.00(2.00, 66.00)	49.29		
	Enterprise	36.00(1.00, 78.00)	54.27		

### 3.5 Negative binomial regression analysis results

The negative binomial regression model’s results, presented in Table 3, indicate that variables related to disease prevention and control themes, positive emotional forms, institutional authorship, and tone type variations demonstrated statistical significance (*p* < 0.05). Conversely, variables associated with healthy lifestyle themes, neutral emotional forms, private authorship, and platform differences did not reach statistical significance (*p* > 0.05). Furthermore, a negative correlation was identified between various platforms, themes, emotional forms, and private authorship, while tone type variations and corporate authorship exhibited a positive correlation.

**Table 3:** Summary of negative binomial regression analysis results.

Parameter	Regression coefficient	Standard error	95% Wald confidence interval		Hypothesis testing		
			Lower limit	Upper limit	Wald’s *χ*^2^	Degrees of freedom	*P*
Intercept	4.387	0.9079	2.608	6.166	23.349	1	0.000
Different platforms							
Video platforms	0.615	0.4209	−0.210	1.439	2.132	1	0.144
WeChat	0.471	0.4968	−0.503	1.445	0.899	1	0.343
Weibo (reference category)							
Different themes							
Healthy lifestyle	−0.289	0.6255	−1.515	0.937	0.214	1	0.644
Disease prevention and control	−1.049	0.3127	−1.662	−0.436	11.257	1	0.001
Traditional Chinese medicine (reference category)							
Different sentence types							
Interrogative sentences	2.143	0.6336	0.901	3.385	11.436	1	0.001
Declarative sentences	1.253	0.5021	0.269	2.237	6.224	1	0.013
Exclamatory sentences	2.386	0.4778	1.449	3.322	24.938	1	0.000
Imperative sentences (reference category)							
Different emotional forms							
Positive	−1.370	0.3802	−2.115	−0.625	12.982	1	0.000
Neutral	−0.439	0.4475	−1.317	0.438	0.964	1	0.326
Negative (reference category)							
Different authors							
Institutional	1.048	0.4973	0.073	2.022	4.440	1	0.035
Private	−0.440	0.3575	−1.140	0.261	1.513	1	0.219
Corporate (reference category)							

## 4 Discussion

Given the complex nature and inherent variability in the dissemination of health science misinformation, the establishment of a dynamic weighting mechanism is crucial. This necessitates the regular collection of data across diverse dissemination scenarios, followed by a rigorous re-evaluation of sensitivity. Based on the subsequent analysis, the weighting of each indicator should be meticulously refined to ensure the accuracy and efficacy of the evaluation system. Beyond considering the sensitivity and interrelationships of each indicator, additional factors can be integrated into the weighting design, including the specific type of misinformation, the characteristics of the dissemination platform, and the attributes of the target audience. Further validation and optimization of these data are recommended, alongside the involvement of a broader cohort of experts in the weighting design and adjustment processes, to enhance the scientific rigor and rationality of the weighting methodology.

A comparative analysis of platform activity revealed that the WeChat platform exhibited superior dissemination capabilities for health-related rumors information. This disparity may be attributed to WeChat’s extensive user base and robust social networking features, where users primarily interact within close-knit networks (13). The high frequency of interactions enhances content propagation efficiency, inadvertently facilitating the spread of misinformation. For instance, the claim “If you do not nourish your heart in winter, all diseases will come to you,” which recommends consuming specific foods for purported cardiovascular and sleep benefits, targets older adults. This type of content leverages traditional Chinese medicine (TCM) concepts, promoting the idea that certain foods or methods can “cure all diseases,” thereby misleading older adults into blindly following such advice. In reality, TCM emphasizes individualized treatment based on diagnosis, and cardiovascular health and sleep quality require comprehensive management (e.g., diet, exercise, emotional regulation), negating the existence of a singular “cure-all” solution. Regulatory bodies and platform operators should enhance their monitoring and oversight of health-related scientific communication on the WeChat platform to mitigate the propagation of misinformation.

Analysis of topic-specific activity demonstrated that the disease prevention theme performed relatively well across sample size, median activity, and rank mean, suggesting its potential dominance in overall activity. For example, the claim “4 vegetables with ‘penicillin’ properties, doctors recommend: eat them frequently in cold weather to boost immunity and reduce illness!” promotes immunity enhancement through disease prevention. However, enhancing immunity should be achieved through balanced nutrition rather than solely relying on specific vegetables. This type of misinformation exaggerates the effects of certain foods and lacks scientific validity. Furthermore, the TCM health preservation theme exhibited considerable variability in activity distribution, possibly due to the complexity of TCM knowledge, making it difficult for the general public to accurately discern truth from falsehood. This creates an environment conducive to the spread of misinformation, where highly active rumors can lead to public adoption of inappropriate health practices, potentially harming their health. The healthy lifestyle theme showed the poorest performance, likely because disease prevention is a key area of public concern, and the dissemination of some rumors is limited due to the dissemination of relevant knowledge and information from official channels. Implementing technical review and authoritative dissemination to rapidly counteract high-activity misinformation, alongside tiered educational interventions customized to subject-specific expertise, will improve public discernment of credible information across various domains, thus fundamentally reducing the proliferation of falsehoods.

A comparative assessment of activity levels across distinct sentence moods reveals that declarative sentences, characterized by a relatively neutral tone, primarily present factual information regarding health, lacking elements that elicit significant emotional responses or behavioral changes in the audience. However, their high frequency may still facilitate dissemination despite the low engagement of individual content. Interrogative sentences exhibit a higher mean rank, while exclamatory sentences demonstrate the highest activity, followed by imperative sentences, indicating potential advantages in attracting attention or triggering interaction. For instance, the exclamatory sentence, “Four medicines are the nemesis of colon cancer, affordable for everyone, ‘breaking down’ cancer cells one by one!” exaggerates the anti-cancer efficacy of traditional Chinese medicine (TCM), disregarding standard medical treatments. In reality, cancer treatment necessitates adherence to evidence-based medicine; TCM can serve as an adjuvant therapy but cannot replace standardized treatments such as surgery, radiotherapy, and chemotherapy. The claim of “curing cancer” lacks scientific substantiation. Utilizing a tone-based methodology, our objective is to achieve “precise interception and cognitive restructuring.” This entails the deployment of an intelligent review system designed to identify and suppress content exhibiting high-risk tonal characteristics. This approach enhances public comprehension of the communicative intent inherent in diverse tones, thereby attenuating the emotional impact and cognitive susceptibilities leveraged by misinformation, and promoting rational discernment.

A comparative analysis of activity levels across different emotional forms indicates that rumors with negative emotional valence, due to their strong stimulation and capacity to induce panic, are the most active type in health science popularization. When individuals encounter content detailing the adverse consequences of a specific disease or the serious hazards of a particular food, they often share this information immediately out of concern for their own and their family’s health to alert those around them (14). From a long-term perspective, frequent exposure to these negative emotional forms of rumors can lead to public anxiety and panic, negatively impacting societal psychological health. Moreover, the rapid and widespread dissemination of these rumors presents significant challenges to debunking efforts. Even with timely debunking, the adverse effects may be difficult to completely mitigate, requiring additional time and resources to restore public confidence and correct health misconceptions. For example, rumors like “Brain death and organ transplantation are a huge lie” often attract attention by creating negativity, panic, and anxiety, but their content usually lacks scientific basis and contradicts existing medical knowledge and practice. Although the overall activity of rumors with positive emotional forms is lower, their potential harm cannot be ignored. These rumors typically attract audiences by exploiting people’s desire for health and a better life (15). To bolster public resilience against emotional mobilization, it is crucial to cultivate a cognitive framework that prioritizes evidence-based decision-making over emotional responses, thereby fundamentally mitigating the susceptibility to emotional manipulation inherent in misinformation campaigns.

A comparative analysis of activity levels across different author types demonstrates that institutional authors exhibit the highest mean rank, indicating superior overall performance in terms of activity. This may be attributed to the inherent authority and credibility of institutions, coupled with their diverse dissemination channels. When institutions release ostensibly professional health science popularization content, the public may be more inclined to believe and disseminate it, even if the content is fallacious. For example, “Eating one dish leads to hepatitis! Many people love this dish.” employs an exaggerated tone to disseminate incorrect health science popularization, causing unnecessary panic. In reality, the direct causal relationship between food and hepatitis requires scientific validation and cannot be established solely based on rumors. Private authors, primarily individual actors, often lack professional knowledge and dissemination resources. The health science popularization rumors they release may be based on personal experience, hearsay, or unverified information, resulting in lower content quality and credibility, thus limiting large-scale dissemination. Compared with institutional and private authors, corporate authors may prioritize their products and brands in their dissemination efforts, resulting in less frequent and less professional health science popularization content (16), consequently yielding lower overall activity. By implementing qualification verification, iterative review processes, and robust accountability mechanisms, we intend to expeditiously mitigate the propagation of misinformation originating from high-risk authors. Moreover, we will institute a tripartite system comprising author self-regulation, platform oversight, and public monitoring. This framework will guide diverse author cohorts in establishing the parameters of responsible communication, thereby severing risk vectors at the source and ultimately fostering a robust information dissemination ecosystem.

The propagation of health misinformation is significantly modulated by platform characteristics, topical focus, tonal nuances, emotional valence, and authorial attributes. Video-sharing platforms demonstrate the most substantial negative coefficient, succeeded by WeChat, neither of which achieved statistical significance. Weibo serves as the reference category; theoretically, its misinformation activity should exceed that of video platforms and WeChat. However, the non-significant findings suggest that the current data are insufficient to definitively establish significant disparities among video platforms, WeChat, and Weibo in their influence on health misinformation activity. Future investigations should further explore platform-specific attributes (e.g., user demographics, content moderation mechanisms, information dissemination models) and other potential influencing factors to more precisely delineate their respective roles in health misinformation activity. This will provide a more robust foundation for formulating targeted misinformation control strategies. Topic analysis reveals that health lifestyle and disease prevention topics exhibit negative regression coefficients, indicating reduced activity compared to Traditional Chinese Medicine (TCM) and wellness topics. This may be attributed to the high demand for specialized information in these areas, which is difficult for general content to satisfy. Additionally, these topics may induce user information fatigue due to frequent discussion. TCM and wellness topics may exhibit higher activity due to the unique cultural and professional aspects of TCM, making them less easily identifiable as misinformation. Tone analysis indicates that exclamatory, declarative, and interrogative tones have negative and significant regression coefficients, suggesting that the activity of misinformation with imperative tones is lower than that of exclamatory, declarative, and interrogative tones. Imperative tones may render information delivery rigid, reducing user interest. Exclamatory and interrogative tones can stimulate curiosity and critical thinking, promoting interaction and increasing activity. Emotional valence analysis reveals that positive valence has a significant negative effect, suggesting that positive tones may inhibit misinformation activity, possibly because positive tones are often associated with authoritative and positive health communication, thereby limiting the spread of misinformation. Neutral tones have a negative coefficient but are not statistically significant, indicating an unclear impact. Negative tones serve as the reference group, suggesting that positive tones should be emphasized in health communication to enhance content credibility and dissemination, thereby suppressing misinformation. Author analysis indicates that institutional authors have a significant positive effect, indicating that if institutional authors are involved in health misinformation, their activity is higher, as their authority facilitates widespread dissemination. Private authors have a negative coefficient but are not statistically significant, indicating no clear association with misinformation activity. Corporate entities serve as the reference group, suggesting the need to strengthen the supervision of institutional authors to ensure the scientific accuracy of their disseminated content and prevent the spread of misinformation through their influence.

## 5 Conclusion

The misinformation addressed in this manuscript predominantly comprises three categories: exaggerated claims regarding disease prevention, unsubstantiated assertions related to traditional Chinese medicine, and extreme recommendations concerning healthy lifestyles. The core issue within these instances is their divergence from established scientific consensus, employing emotional appeals, rhetorical techniques, or appeals to authority to misinform the public. This study, through the presentation of specific misinformation cases and tiered prevention strategies, aims to improve public awareness of the risks associated with misinformation while offering actionable solutions for governance bodies. Future research should prioritize the categorization of misinformation based on societal impact, focusing on strategies to enhance public resilience to misinformation and the development of a comprehensive prevention framework. This would facilitate the establishment of a multi-faceted governance structure incorporating “platform technology interception, science communication education, and misinformation source management,” including dynamic labeling (e.g., real-time annotation of “suspected false information”), international collaboration, and the co-creation of multilingual datasets (17). Moreover, adherence to the principles of “source verification, distinguishing between emotion and fact, and consulting professional guidelines” is essential to mitigate susceptibility to singular information or extreme claims.

## Data Availability

The raw data supporting the conclusions of this article will be made available by the authors, without undue reservation.
