# Supplementary material for: An analysis of the factors influencing engagement metrics within the dissemination of health science misinformation
Source: Front Public Health. 2025 Jun 6;13:1571210. doi: 10.3389/fpubh.2025.1571210 (PMC12180413; doi:10.3389/fpubh.2025.1571210)
Supplement: Supplementary file 1 [file Data_Sheet_1.pdf]

Expert interviews on the weighting of four indicators related to health misinformation dissemination

To determine the weighting of four indicators relevant to the dissemination of misinformation in health science popularization, we conducted expert interviews. Initially, the research team identified three key characteristics of such misinformation: scientific inaccuracy, high dissemination potential, and significant risk. A dataset comprising 109 instances of health science popularization misinformation, collected and screened from November 1 to November 30, 2024, was analyzed. The subsequent analysis focused on activity, guided by the Uses and Gratifications Theory, Social Presence Theory, Six Degrees of Separation Theory, and Communication Effects Theory. Four indicators were selected: the number of reads, comments, shares, and likes. Experts were tasked with assigning weights to these indicators and providing rationales.

Expert 1 prioritized reads, shares, likes, and comments, with reads deemed most critical. The expert noted a correlation between reads, shares, and likes, necessitating correction within any function incorporating these indicators. The expert also highlighted the challenges in evaluating comments due to their varied valence, suggesting that a quantitative approach alone could introduce bias.

Expert 2 proposed a weighting order of shares > likes > comments > reads. Shares were considered indicative of active information dissemination, aligning with the Six Degrees of Separation Theory, and were thus weighted highest. Likes, reflecting user engagement and content attractiveness, were ranked second. Comments, representing user interaction and discussion, were weighted third. Reads, representing initial information contact, were deemed to have the weakest impact on activity.

Expert 3 recommended a weighting of shares (40%) > reads (30%) > comments (20%) > likes (10%). Shares were identified as the primary vector for misinformation penetration into high-risk groups, warranting the highest weight. Reads were considered second in importance, followed by comments, with likes receiving the lowest weight.

Expert 4 proposed a weighting order of shares, reads, comments, and likes. Shares, directly expanding the dissemination scope, were weighted first. Reads, representing the basis of information contact, were ranked second. Comments, reflecting user interaction, were ranked third, while likes were weighted last.

Expert 5 suggested a weighting of shares, reads, likes, and comments. Shares were identified as the core behavior in cross-circle dissemination, warranting the highest weight. Reads were considered a direct manifestation of users actively obtaining information, followed by likes, and comments.
