# Supplementary material for: An analysis of the factors influencing engagement metrics within the dissemination of health science misinformation
Source: Front Public Health. 2025 Jun 6;13:1571210. doi: 10.3389/fpubh.2025.1571210 (PMC12180413; doi:10.3389/fpubh.2025.1571210)
Supplement: Supplementary file 2 [file Data_Sheet_2.pdf]

| 平台 | 平台编码 | 标题 | 标题编码 | 句型 | 句型编码 | 语气 | 语气编码 | 作者 |
|---|---|---|---|---|---|---|---|---|
| 短视频 | 1 | 中医养生 | 3 | 疑问句 | 1 | 积极 | 1 | 机构 |
| 抖音 | 1 | 健康生活方式 | 1 | 陈述句 | 2 | 消极 | 3 | 个人 |
| 微信 | 2 | 疾病防控 | 2 | 感叹句 | 3 | 消极 | 3 | 机构 |
| 微信 | 2 | 中医养生 | 3 | 陈述句 | 2 | 积极 | 1 | 企业 |
| 微信 | 2 | 疾病防控 | 2 | 感叹句 | 3 | 消极 | 3 | 企业 |
| 微信 | 2 | 中医养生 | 3 | 陈述句 | 2 | 积极 | 1 | 个人 |
| 微信 | 2 | 中医养生 | 3 | 陈述句 | 2 | 积极 | 1 | 机构 |
| 微信 | 2 | 疾病防控 | 2 | 陈述句 | 2 | 消极 | 3 | 个人 |
| 微信 | 2 | 疾病防控 | 2 | 感叹句 | 3 | 中性 | 2 | 个人 |
| 微信 | 2 | 疾病防控 | 2 | 祈使句 | 4 | 消极 | 3 | 机构 |
| 微信 | 2 | 中医养生 | 3 | 陈述句 | 2 | 积极 | 1 | 个人 |
| 微信 | 2 | 疾病防控 | 2 | 陈述句 | 2 | 中性 | 2 | 企业 |
| 微信 | 2 | 疾病防控 | 2 | 感叹句 | 3 | 消极 | 3 | 个人 |
| 微信 | 2 | 疾病防控 | 2 | 陈述句 | 2 | 消极 | 3 | 机构 |
| 微信 | 2 | 中医养生 | 3 | 陈述句 | 2 | 中性 | 2 | 个人 |
| 视频网站 | 1 | 中医养生 | 3 | 感叹句 | 3 | 中性 | 2 | 个人 |
| 视频网站 | 1 | 疾病防控 | 2 | 陈述句 | 2 | 积极 | 1 | 个人 |
| 微信 | 2 | 中医养生 | 3 | 感叹句 | 3 | 积极 | 1 | 企业 |
| 视频网站 | 1 | 疾病防控 | 2 | 陈述句 | 2 | 积极 | 1 | 个人 |
| 微信 | 2 | 疾病防控 | 2 | 疑问句 | 1 | 消极 | 3 | 个人 |
| 微信 | 2 | 中医养生 | 3 | 陈述句 | 2 | 积极 | 1 | 机构 |
| 视频网站 | 1 | 健康生活方式 | 1 | 陈述句 | 2 | 消极 | 3 | 个人 |
| 微信 | 2 | 中医养生 | 3 | 陈述句 | 2 | 中性 | 2 | 个人 |
| 微信 | 2 | 中医养生 | 3 | 感叹句 | 3 | 中性 | 2 | 机构 |
| 微信 | 2 | 健康生活方式 | 1 | 祈使句 | 4 | 消极 | 3 | 个人 |
| 微信 | 2 | 疾病防控 | 2 | 感叹句 | 3 | 消极 | 3 | 企业 |
| 微信 | 2 | 疾病防控 | 2 | 陈述句 | 2 | 消极 | 3 | 机构 |
| 微信 | 2 | 疾病防控 | 2 | 感叹句 | 3 | 积极 | 1 | 机构 |
| 微信 | 2 | 疾病防控 | 2 | 祈使句 | 4 | 消极 | 3 | 机构 |
| 微信 | 2 | 疾病防控 | 2 | 陈述句 | 2 | 中性 | 2 | 机构 |
| 微信 | 2 | 疾病防控 | 2 | 感叹句 | 3 | 消极 | 3 | 个人 |
| 微信 | 2 | 疾病防控 | 2 | 疑问句 | 1 | 中性 | 2 | 机构 |
| 微信 | 2 | 疾病防控 | 2 | 疑问句 | 1 | 消极 | 3 | 机构 |
| 视频网站 | 2 | 疾病防控 | 2 | 陈述句 | 2 | 消极 | 3 | 企业 |
| 视频网站 | 1 | 疾病防控 | 2 | 感叹句 | 3 | 中性 | 2 | 个人 |
| 微信 | 2 | 疾病防控 | 2 | 陈述句 | 2 | 消极 | 3 | 机构 |
| 微信 | 2 | 疾病防控 | 2 | 陈述句 | 2 | 消极 | 3 | 个人 |
| 微信 | 2 | 疾病防控 | 2 | 陈述句 | 2 | 中性 | 2 | 个人 |
| 微信 | 2 | 疾病防控 | 2 | 感叹句 | 3 | 消极 | 3 | 个人 |
| 微信 | 2 | 疾病防控 | 2 | 陈述句 | 2 | 中性 | 2 | 企业 |
| 微信 | 2 | 疾病防控 | 2 | 陈述句 | 2 | 积极 | 1 | 机构 |
| 视频网站 | 1 | 疾病防控 | 2 | 陈述句 | 2 | 消极 | 3 | 个人 |
| 视频网站 | 1 | 疾病防控 | 2 | 祈使句 | 4 | 中性 | 2 | 机构 |
| 视频网站 | 1 | 疾病防控 | 2 | 陈述句 | 2 | 消极 | 3 | 机构 |
| 视频网站 | 1 | 疾病防控 | 2 | 陈述句 | 2 | 中性 | 2 | 个人 |
| 微信 | 2 | 健康生活方式 | 1 | 陈述句 | 2 | 消极 | 3 | 个人 |
| 微信 | 2 | 中医养生 | 3 | 陈述句 | 2 | 中性 | 2 | 个人 |

| | | | | | | | | |
|---|---|---|---|---|---|---|---|---|
| 微信 | 2 | 疾病防控 | 2 | 疑问句 | 1 | 消极 | 3 | 机构 |
| 微信 | 2 | 中医养生 | 3 | 感叹句 | 3 | 中性 | 2 | 企业 |
| 视频网站 | 1 | 疾病防控 | 2 | 感叹句 | 3 | 积极 | 1 | 个人 |
| 视频网站 | 1 | 疾病防控 | 2 | 陈述句 | 2 | 积极 | 1 | 个人 |
| 视频网站 | 1 | 疾病防控 | 2 | 陈述句 | 2 | 消极 | 3 | 个人 |
| 视频网站 | 1 | 疾病防控 | 2 | 感叹句 | 3 | 积极 | 1 | 个人 |
| 视频网站 | 1 | 健康生活方式 | 1 | 陈述句 | 2 | 中性 | 2 | 个人 |
| 视频网站 | 1 | 中医养生 | 3 | 陈述句 | 2 | 中性 | 2 | 个人 |
| 视频网站 | 1 | 疾病防控 | 2 | 陈述句 | 2 | 消极 | 3 | 个人 |
| 视频网站 | 1 | 疾病防控 | 2 | 陈述句 | 2 | 积极 | 1 | 个人 |
| 视频网站 | 1 | 中医养生 | 3 | 感叹句 | 3 | 中性 | 2 | 机构 |
| 视频网站 | 1 | 中医养生 | 3 | 感叹句 | 3 | 中性 | 2 | 机构 |
| 视频网站 | 1 | 中医养生 | 3 | 陈述句 | 2 | 消极 | 3 | 机构 |
| 视频网站 | 1 | 中医养生 | 3 | 疑问句 | 1 | 消极 | 3 | 机构 |
| 视频网站 | 1 | 中医养生 | 3 | 感叹句 | 3 | 积极 | 1 | 个人 |
| 微博 | 3 | 中医养生 | 3 | 陈述句 | 2 | 积极 | 1 | 个人 |
| 微博 | 3 | 中医养生 | 3 | 感叹句 | 3 | 积极 | 1 | 企业 |
| 微博 | 3 | 中医养生 | 3 | 祈使句 | 4 | 积极 | 1 | 企业 |
| 微博 | 3 | 健康生活方式 | 1 | 陈述句 | 2 | 中性 | 2 | 机构 |
| 视频网站 | 1 | 中医养生 | 3 | 陈述句 | 2 | 中性 | 2 | 企业 |
| 视频网站 | 1 | 中医养生 | 3 | 陈述句 | 2 | 积极 | 1 | 个人 |
| 视频网站 | 1 | 中医养生 | 3 | 陈述句 | 2 | 积极 | 1 | 个人 |
| 视频网站 | 1 | 中医养生 | 3 | 疑问句 | 1 | 积极 | 1 | 个人 |
| 微博 | 3 | 健康生活方式 | 1 | 陈述句 | 2 | 中性 | 2 | 企业 |
| 视频网站 | 1 | 健康生活方式 | 1 | 陈述句 | 2 | 积极 | 1 | 个人 |
| 视频网站 | 1 | 健康生活方式 | 1 | 陈述句 | 2 | 积极 | 1 | 个人 |
| 视频网站 | 1 | 中医养生 | 3 | 陈述句 | 2 | 积极 | 1 | 个人 |
| 视频网站 | 1 | 中医养生 | 3 | 陈述句 | 2 | 中性 | 2 | 个人 |
| 视频网站 | 1 | 中医养生 | 3 | 陈述句 | 2 | 积极 | 1 | 个人 |
| 视频网站 | 1 | 中医养生 | 3 | 陈述句 | 2 | 中性 | 2 | 个人 |
| 视频网站 | 1 | 中医养生 | 3 | 陈述句 | 2 | 中性 | 2 | 企业 |
| 视频网站 | 1 | 疾病防控 | 2 | 感叹句 | 3 | 积极 | 1 | 企业 |
| 视频网站 | 1 | 中医养生 | 3 | 感叹句 | 3 | 积极 | 1 | 个人 |
| 视频网站 | 1 | 中医养生 | 3 | 感叹句 | 3 | 积极 | 1 | 个人 |
| 视频网站 | 1 | 中医养生 | 3 | 陈述句 | 2 | 中性 | 2 | 个人 |
| 视频网站 | 1 | 中医养生 | 3 | 陈述句 | 2 | 中性 | 2 | 个人 |
| 视频网站 | 1 | 中医养生 | 3 | 陈述句 | 2 | 中性 | 2 | 个人 |
| 视频网站 | 1 | 疾病防控 | 2 | 祈使句 | 4 | 中性 | 2 | 个人 |
| 微信 | 2 | 中医养生 | 3 | 感叹句 | 3 | 消极 | 3 | 企业 |
| 微信 | 2 | 中医养生 | 3 | 感叹句 | 3 | 积极 | 1 | 企业 |
| 微信 | 2 | 疾病防控 | 2 | 感叹句 | 3 | 积极 | 1 | 企业 |
| 微信 | 2 | 疾病防控 | 2 | 感叹句 | 3 | 消极 | 3 | 企业 |
| 微信 | 2 | 疾病防控 | 2 | 感叹句 | 3 | 积极 | 1 | 个人 |

| | | | | | | | | |
|---|---|---|---|---|---|---|---|---|
| 微信 | 2 | 疾病防控 | 2 | 感叹句 | 3 | 消极 | 3 | 机构 |
| 视频网站 | 1 | 疾病防控 | 2 | 感叹句 | 3 | 积极 | 1 | 个人 |
| 微信 | 2 | 健康生活方式 | 1 | 疑问句 | 1 | 积极 | 1 | 企业 |
| 微信 | 2 | 疾病防控 | 2 | 感叹句 | 3 | 积极 | 1 | 个人 |
| 微信 | 2 | 疾病防控 | 2 | 陈述句 | 2 | 消极 | 3 | 企业 |
| 微信 | 2 | 疾病防控 | 2 | 疑问句 | 1 | 积极 | 1 | 企业 |
| 微信 | 2 | 疾病防控 | 2 | 感叹句 | 3 | 中性 | 2 | 企业 |
| 微博 | 3 | 疾病防控 | 2 | 陈述句 | 2 | 中性 | 2 | 个人 |
| 微博 | 3 | 疾病防控 | 2 | 感叹句 | 3 | 积极 | 1 | 个人 |
| 微博 | 3 | 疾病防控 | 2 | 陈述句 | 2 | 中性 | 2 | 企业 |
| 微博 | 3 | 疾病防控 | 2 | 陈述句 | 2 | 积极 | 1 | 个人 |
| 微博 | 3 | 疾病防控 | 2 | 感叹句 | 3 | 积极 | 1 | 个人 |
| 微博 | 3 | 疾病防控 | 2 | 陈述句 | 2 | 中性 | 2 | 个人 |
| 微博 | 3 | 疾病防控 | 2 | 陈述句 | 2 | 中性 | 2 | 个人 |
| 微博 | 3 | 疾病防控 | 2 | 感叹句 | 3 | 积极 | 1 | 个人 |
| 微博 | 3 | 疾病防控 | 2 | 陈述句 | 2 | 中性 | 2 | 企业 |
| 微博 | 3 | 疾病防控 | 2 | 陈述句 | 2 | 中性 | 2 | 个人 |
| 微博 | 3 | 中医养生 | 3 | 陈述句 | 2 | 积极 | 1 | 个人 |
| 微博 | 3 | 中医养生 | 3 | 陈述句 | 2 | 中性 | 2 | 个人 |

| 作者编码 | 1天活跃度 |
|---|---|
| 1 | 3192 |
| 2 | 2152 |
| 1 | 1938 |
| 3 | 397 |
| 3 | 217 |
| 2 | 119 |
| 1 | 135 |
| 2 | 91 |
| 2 | 239 |
| 1 | 137 |
| 2 | 102 |
| 3 | 58 |
| 2 | 58 |
| 1 | 139 |
| 2 | 68 |
| 2 | 51 |
| 2 | 168 |
| 3 | 40 |
| 2 | 66 |
| 2 | 166 |
| 1 | 86 |
| 2 | 41 |
| 2 | 56 |
| 1 | 51 |
| 2 | 58 |
| 3 | 58 |
| 1 | 35 |
| 1 | 31 |
| 1 | 21 |
| 1 | 23 |
| 2 | 77 |
| 1 | 42 |
| 1 | 20 |
| 3 | 21 |
| 2 | 41 |
| 1 | 40 |
| 2 | 22 |
| 2 | 14 |
| 2 | 103 |
| 3 | 11 |
| 1 | 10 |
| 2 | 12 |
| 1 | 31 |
| 1 | 11 |
| 2 | 22 |
| 2 | 14 |
| 2 | 9 |

| | |
|---|---|
| 1 | 10 |
| 3 | 22 |
| 2 | 55 |
| 2 | 34 |
| 2 | 7 |
| 2 | 8 |
| 2 | 6 |
| 2 | 36 |
| 2 | 6 |
| 2 | 5 |
| 1 | 11014 |
| 1 | 32379 |
| 1 | 1739 |
| 1 | 722 |
| 2 | 4 |
| 2 | 2 |
| 3 | 2 |
| 3 | 1 |
| 1 | 2 |
| 3 | 1 |
| 2 | 1 |
| 2 | 1 |
| 2 | 1 |
| 3 | 1 |
| 2 | 1 |
| 2 | 1 |
| 2 | 1 |
| 2 | 1 |
| 2 | 2 |
| 2 | 1 |
| 3 | 1 |
| 3 | 1 |
| 2 | 2 |
| 2 | 1 |
| 2 | 1 |
| 2 | 1 |
| 2 | 1 |
| 2 | 76 |
| 3 | 333 |
| 3 | 68 |
| 3 | 66 |
| 3 | 55 |
| 2 | 39 |

| | |
|---|---|
| 1 | 22 |
| 2 | 8 |
| 3 | 32 |
| 2 | 964 |
| 3 | 2047 |
| 3 | 433 |
| 3 | 88 |
| 2 | 3 |
| 2 | 6 |
| 3 | 1 |
| 2 | 1 |
| 2 | 4 |
| 2 | 16 |
| 2 | 40 |
| 2 | 7 |
| 3 | 1 |
| 2 | 136 |
| 2 | 83 |
| 2 | 1142 |
